# Functional Magnetic Resonance Imaging in Acute Kidney Injury: Present Status

**DOI:** 10.1155/2016/2027370

**Published:** 2016-01-26

**Authors:** Hai Ying Zhou, Tian Wu Chen, Xiao Ming Zhang

**Affiliations:** Sichuan Key Laboratory of Medical Imaging, Department of Radiology, Affiliated Hospital of North Sichuan Medical College, 63 Wenhua Road, Shunqing District, Nanchong, Sichuan 637000, China

## Abstract

Acute kidney injury (AKI) is a common complication of hospitalization that is characterized by a sudden loss of renal excretory function and associated with the subsequent development of chronic kidney disease, poor prognosis, and increased mortality. Although the pathophysiology of renal functional impairment in the setting of AKI remains poorly understood, previous studies have identified changes in renal hemodynamics, perfusion, and oxygenation as key factors in the development and progression of AKI. The early assessment of these changes remains a challenge. Many established approaches are not applicable to humans because of their invasiveness. Functional renal magnetic resonance (MR) imaging offers an alternative assessment tool that could be used to evaluate renal morphology and function noninvasively and simultaneously. Thus, the purpose of this review is to illustrate the principle, application, and role of the techniques of functional renal MR imaging, including blood oxygen level-dependent imaging, arterial spin labeling, and diffusion-weighted MR imaging, in the management of AKI. The use of gadolinium in MR imaging may exacerbate renal impairment and cause nephrogenic systemic fibrosis. Therefore, dynamic contrast-enhanced MR imaging will not be discussed in this paper.

## 1. Introduction

Acute kidney injury (AKI) is a common complication of hospitalization that occurs in various clinical settings, particularly in the setting of critical illness. It is characterized by a sudden loss of renal excretory function and associated with the subsequent development of chronic kidney disease, poor prognosis, and increased mortality. A variety of causes, such as renal ischemic events, exposure to nephrotoxic substances, acute tubular necrosis (ATN), and acute upper urinary tract obstruction, can trigger AKI [1, 2]. Although previous studies have identified changes in renal hemodynamics, perfusion, and oxygenation as key factors in the development and progression of AKI, the pathophysiology of renal functional impairment in the setting of AKI remains poorly understood [3–5]. In clinical practice, the serum creatinine (sCr) levels are attractive for the routine diagnosis and staging of AKI due to the relative simplicity and convenience of the test. However, the sCr level has major limitations as a biomarker for AKI [6]. First, it does not change until approximately 50% of kidney function is lost. Therefore, it is not sensitive to the rapid changes in renal function induced by AKI. Moreover, the lag time between renal injury and the increase in the sCr level results in missed therapeutic opportunities, which may be responsible for the high mortality associated with AKI. Second, the sCr level depends on many other factors, such as muscle mass, age, sex, medications, and hydration status. Thus, a better understanding and early detection of AKI are important for its treatment.

With the development of magnetic resonance (MR) imaging, functional renal MR imaging has rapidly grown and could be used to evaluate renal morphology and function noninvasively and simultaneously [7, 8]. The main MR imaging techniques include blood oxygen level-dependent (BOLD) imaging, arterial spin labeling (ASL), dynamic contrast-enhanced MR imaging (DCE-MRI), diffusion-weighted imaging (DWI), intravoxel incoherent motion (IVIM), diffusion tensor imaging (DTI), and diffusion kurtosis imaging (DKI). These approaches can provide information on intrarenal oxygenation, perfusion, and diffusion on a microstructural level, which may not only allow the noninvasive detection of the presence and severity of renal abnormalities associated with AKI in preclinical setting, but also demonstrate the pathophysiology and progress of AKI. Because the use of gadolinium in MR imaging may exacerbate renal impairment and cause nephrogenic systemic fibrosis [9], DCE-MRI will not be discussed in this paper. Thus, the objective of this paper is to provide a brief overview of the principle, application, and role of the remaining techniques in the management of AKI.

## 2. BOLD MR Imaging

The pathophysiology of AKI is not yet fully understood, but renal tissue hypoperfusion and hypoxia are well accepted to be closely related to the pathophysiology of all forms of AKI [5]. The direct measurement of oxygen partial pressure (*p*O_2_) by oxygen-sensing electrodes that penetrate the renal parenchyma remains the gold standard for assessing renal tissue oxygenation. However, this measurement technique is invasive and highly complex, which makes it not applicable for widespread use [10, 11]. Therefore, a noninvasive approach to assess renal oxygenation status in vivo is needed.

**Figure 1:**
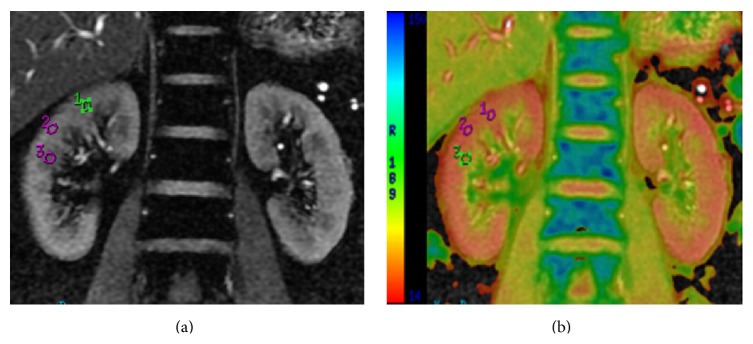
Representative mGRE images obtained from a healthy volunteer. (a) An mGRE image with long TE is used to place regions of interest (ROIs) on the medulla and cortex. Regions of cortex appear bright with high signal intensity, whereas medullary pyramids appear darker with a low signal intensity on the mGRE image. (b) The *R*2^*∗*^ map is from the same slice position, which was scaled from 14 (red) to 150 (blue), representing a range of *R*2^*∗*^ levels. Larger *R*2^*∗*^ values correspond to higher levels of hypoxia. In this map, the medulla can be clearly distinguished from the cortex because the medulla has a higher *R*2^*∗*^ value or a colder color in the *R*2^*∗*^ map.

In the mid 1990s, BOLD MR imaging was demonstrated as an important noninvasive technique to assess intrarenal oxygenation under physiologic and pathophysiologic conditions in experimental animals and humans [8, 12–14]. The paramagnetic properties of deoxyhemoglobin are utilized as an endogenous marker to acquire images to measure tissue oxygenation. Specifically, increased deoxyhemoglobin concentrations change the magnetic spin properties of neighboring water molecules, which accelerates magnetic spin dephasing and decreases the signal intensity on apparent spin-spin relaxation time-weighted (*T*2^*∗*^) MR images. The rate of magnetic spin dephasing, *R*2^*∗*^( = 1/*T*2^*∗*^), has been used as a quantitative parameter to reflect renal oxygenation (Figure 1). An increase in *R*2^*∗*^ implies an increased deoxyhemoglobin concentration and decreased tissue* p*O_2_, which may result from impaired renal perfusion, decreased blood O_2_ content, or increased O_2_ consumption [12]. A strong correlation has been proved between renal BOLD MRI to tissue oxygen partial pressure (*p*O_2_) [15].

Recently, BOLD has been widely used to study intrarenal oxygenation in human and animal studies of AKI [8, 13, 14]. BOLD MR imaging was demonstrated to effectively detect changes in intrarenal oxygenation by measuring the *R*2^*∗*^ levels of the renal cortex and medulla. In pig models of AKI induced by acute renal ischemia, the *R*2^*∗*^ values of the cortex and medulla both increased, which demonstrated a reduction in intrarenal oxygenation in parallel with decreased intrarenal blood flow during acute ischemia. After reperfusion, the intrarenal oxygenation levels immediately returned to baseline oxygenation, which demonstrated that some of the early changes in renal oxygenation due to AKI may reverse [16, 17]. Furthermore, the degree of ischemic reperfusion injury commonly influences the recovery of renal function [18].

BOLD MRI has also been utilized to study the mechanisms of contrast-induced AKI (CIAKI). It is indicated that the administration of contrast agent caused an early and transient decrease in the medullary *R*2^*∗*^ followed by a sustained increase above the baseline in animal models of contrast-induced AKI (CIAKI), whereas minimal changes were observed in the renal cortex [19, 20]. The differences in the variations in *R*2^*∗*^ between the renal medullary and cortex agree with the basis of renal pathophysiology. Specifically, most of the oxygen consumed by the kidney is due to the reabsorption of filtered sodium by the medullary thick ascending limb of the loop of Henle, but only approximately 5% of renal blood flow is supplied to the medulla, which makes it more susceptible to hypoperfusion and hypoxia. However, conflicting mechanisms of the initial decrease in the medullary *R*2^*∗*^ after contrast agent injection have been reported, and a consensus has not been reached. Arakelyan et al. [21] attributed this change to an increase in renal tubular volume due to dye-induced osmotic diuresis, which decreased the blood volume fraction. Conversely, other investigators demonstrated that this change may be due to an initial increase in medullary blood flow. Li et al. [20] used 4 contrast media with different physicochemical properties to assess the differences in intrarenal oxygenation in CIAKI-susceptible rats by BOLD MR imaging. They also demonstrated that the immediate increase in *R*2^*∗*^ in the renal inner stripe of the outer medulla (ISOM) after the injection of contrast agent may be the earliest biomarker of AKI. Furthermore, the different viscosities of the contrast agents may lead to a difference in the *R*2^*∗*^ level in the renal cortex and medulla after injection. In addition, the effects of some interventions to mitigate the adverse effects of contrast media have been evaluated using BOLD MRI, and the results showed that the rate of increase in *R*2^*∗*^ in the renal ISOM can be reduced by treatment with furosemide (diuretic) or N-acetylcysteine (NAC; antioxidant) before contrast media injection, but the optimum dose of furosemide and NAC for mitigating the negative effects of contrast media has not yet been determined [22].

Renal oxygenation in renal allografts with ATN has also been studied using BOLD MR imaging, but the current data are controversial and difficult to interpret [23–25]. The *R*2^*∗*^ values of the cortex and medulla reportedly increased in allografts with ATN compared with normally functioning allografts, which suggested decreased oxygen bioavailability both in the cortex and medulla in allografts with ATN [23]. Conversely, Djamali et al. [24] found that the medulla *R*2^*∗*^ levels of ATN allografts decreased compared with normally functioning allografts, which reflected a significant increase in the medullary oxygen bioavailability in ATN allografts. In addition, Sadowski et al. [25] reported that the *R*2^*∗*^ levels of the cortex and medulla did not significantly differ between the ATN group and the normal functioning allografts.

Renal oxygenation in AKI due to other causes, such as sepsis-associated AKI and other nephrotoxin-induced AKI, has also been studied in several experimental animal models and humans using BOLD MRI [26, 27]. However, the number of studies is small, and unifying conclusions or significant insights are lacking. Therefore, this aspect will not be discussed in this review.

In conclusion, BOLD MR imaging not only can noninvasively assess changes in renal oxygenation due to AKI by measuring the *R*2^*∗*^ levels of the renal cortex and medulla, but also can investigate the role of hypoxia in the pathogenesis and progress of AKI. In recent years, this strategy has been widely used to assess AKI. However, further studies are necessary to establish the cut-off *R*2^*∗*^ values for the diagnosis of AKI and evaluate the specificity of *R*2^*∗*^ for the renal oxygenation status.

## 3. ASL

**Figure 2:**
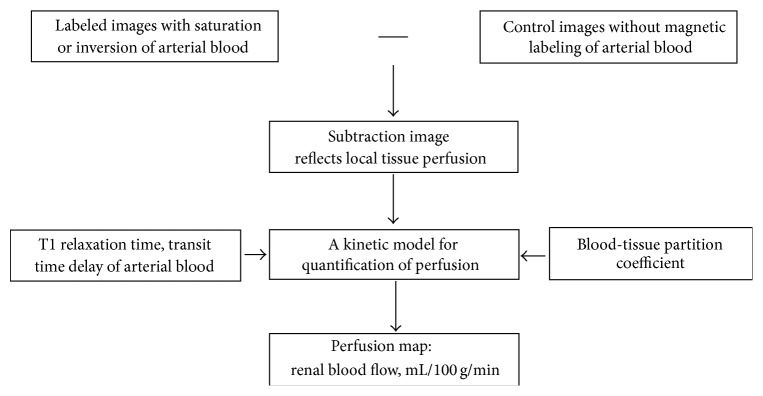
Schematic diagram of ASL imaging. Knowledge of parameters, such as the tissue T1 relaxation time, blood-tissue partition coefficient, and transit time of the blood water to tissue water, is generally required to quantify the perfusion.

ASL is a novel, noninvasive MRI technique to measure tissue perfusion, that is, tissue blood flow [28], by magnetically labeling water protons in the blood as an endogenous contrast agent. First, the water in the blood is labeled before it enters the tissue of interest. The labeled water then flows into tissue and is exchanged with tissue water, thereby altering its magnetization. The perfusion-weighted image is obtained by subtracting the labeled image from a control image with unlabeled blood water to obtain the difference, and the signal intensity is proportional to perfusion. Finally, a kinetic model is used to directly quantify perfusion if other parameters, such as the tissue T1 relaxation time, blood-tissue partition coefficient, and transit time of the blood water to tissue water, are known (Figure 2). Recently, this technique has been widely used to evaluate cerebral perfusion [29]. With respect to the kidney, published studies demonstrated that this approach is another ideal candidate for ASL imaging due to its high physiologic perfusion (both kidneys, which results in approximately 0.5% of the total body mass receiving approximately 25% of the cardiac output) and the perfusion difference between the cortex and medulla (the cortex receives approximately 92~94% of the renal blood flow, whereas only approximately 5% of this flow is supplied to the medulla). The feasibility of flow-sensitive alternating inversion recovery (FAIR) perfusion preparation with a steady-state free precession (True-FISP) ASL quantification of renal perfusion has also been demonstrated in both healthy and disease states [30].

Dong et al. [31] performed a pilot study to demonstrate the feasibility of ASL perfusion MRI in the detection of AKI and found that the cortical, medullary, and global kidney blood flows were significantly lower in AKI patients than in healthy volunteers. This finding suggested that the decrease in renal perfusion is critical to the pathophysiology of AKI which is in agreement with previous reports on the basis of the evaluation of renal blood flow of AKI [3–5]. Furthermore, ASL was also shown to be able to noninvasively detect the severity of AKI and monitor renal perfusion impairment over time in a mouse model of ischemia-induced AKI. The degree of perfusion impairment measured using ASL is related to kidney volume loss, the severity of histopathologic alterations of renal tissue, and the impairment of renal function. In addition, renal perfusion measured by means of ASL may also serve as a noninvasive biomarker to predict the extent of subsequent histologic alterations of the kidney early after the organ is damaged. Thus, ASL may be very valuable for the clinical follow-up of patients who are at risk for AKI and for drug development in experimental renal disease models [32].

Zimmer et al. [33] reported that ASL is a valid alternative to DCE-MRI, and ASL might be preferred for patients with impaired kidney function because the injection of Gd-based contrast agents may exacerbate renal impairment and cause nephrogenic systemic fibrosis. In addition, Chen et al. [34] used ASL and BOLD MRI to evaluate the damage to renal function in CIAKI rats at 3T and found that ASL combined with BOLD can further identify the primary cause of the decrease in renal oxygenation in CIAKI. Compared with BOLD, ASL perfusion MRI can absolutely quantify a well characterized physiological parameter, whereas the quantified parameter obtained by BOLD imaging is a result of complex interactions among renal blood flow, renal blood volume, and oxygen consumption.

However, the relatively low signal-to-noise ratio (SNR) and short signal decay rate of the ASL technique will delay its clinical application. A high-field MR scanner is necessary to enhance the image quality and provide a more accurate analysis of renal perfusion using the ASL technique [35].

## 4. Diffusion-Weighted Imaging

DWI is a powerful technique that provides information on the renal microstructure and function by characterizing water motion on a molecular level [7, 36]. The apparent diffusion coefficient (ADC) is utilized as a quantitative parameter of diffusion, which is calculated from DW images with a monoexponential decay model. Structural changes, such as interstitial fibrosis or tubular atrophy, could result in a decrease in the ADC value, which has been demonstrated to correlate with renal function. Renal diffusion in both healthy and disease states has been evaluated using this technique [7]. In a mouse model of ischemia-induced AKI, the ability of the DWI value to characterize acute and chronic pathology after unilateral AKI was investigated. The ADC value of the renal medulla was shown to be significantly decreased at every time point after AKI, and the renal ADC values changed with the severity of AKI and the degree of interstitial renal fibrosis 4 weeks after AKI. This finding suggested that the decrease in renal diffusion is critical to the pathophysiology of AKI which is associated with renal tissue edema, inflammatory cell infiltration, and subsequent development of interstitial renal fibrosis and tubular atrophy [37].

Nevertheless, the ADC values, which derive from the conventional monoexponential model, provide a mix of information on capillary perfusion and water diffusion in the extravascular space [38]. The accurate diffusion of water molecules, which is considered as a result of altered tissue structures, is difficult to calculate.

Thus, the intravoxel incoherent motion (IVIM) biexponential model of postprocessing was developed. This model allows the pure diffusion and perfusion-dependent diffusion to be differentiated by calculating quantitative parameters using multi-*b*-value DWI [38, 39]. At low *b*-values (*b* < 200 s/mm^2^), the intravoxel spin dephasing caused by the pseudorandom blood flow in the presence of a diffusion gradient will contribute more to signal attenuation, leading to the dependence of the ADC values on perfusion effects. Conversely, at high *b*-values (*b* > 400 s/mm^2^), diffusion attenuation will primarily be due to molecular water diffusion because the blood signal will be mostly suppressed by the large diffusion gradients. Thus, the effects of pseudodiffusion can be excluded to yield the true diffusion measurement. Quantitative parameters, including pure molecular diffusion (*D*), which is closely related to the abnormal biophysical processes, the volume fraction (*f*) of diffusion, which is sensitive to renal fluid loading, and perfusion-related diffusion (*D* ^*∗*^), which is linked to arteriolar vasoconstriction or vasodilation, are simultaneously measured by IVIM MR imaging to obtain information about microvascular dynamics and renal fluid loading.

The potential clinical applications of IVIM in renal lesions have been demonstrated in several pilot studies [40, 41], but AKI has rarely been assessed using IVIM. Liang et al. [42] evaluated pathophysiological alterations in a CIAKI animal model using IVIM. The study demonstrated that IVIM can provide useful information to noninvasively evaluate renal pathophysiological processes in a CIAKI model in vivo. Large-scale animal and human clinical studies should be performed in the future to assess the use of IVIM in AKI due to other causes.

The studies mentioned above all assume that diffusion in the kidney is isotropic. In fact, due to the radial distribution characteristics of the important anatomic structures of the kidney, like vessels and tubules, diffusion is anisotropic and should be assessed by another DWI technique, diffusion tensor imaging (DTI) [43]. This technique not only evaluates the intensity of water diffusion in the kidney but also the locally preferred direction of this diffusion by analyzing multiple measurements with six diffusion gradient directions. This information is used to calculate the pixel-per-pixel fractional anisotropy (FA) map and FA value, which is a dimensional parameter quantifying the amount of diffusion anisotropy within a region of interest. Respiratory motion has been identified as a key factor that limits the application of DTI in abdominal organs, but the use of breath-hold sequences and respiratory triggering has yielded higher quality images with acceptable acquisition times. Thus, DTI has become popular in renal studies [44].

Obvious diffusion anisotropy in the renal medulla has been demonstrated by DTI [45], and decreases in the FA of the medulla are observed in animal models of ischemia-induced AKI [46]. Although the biophysics underlying this anisotropy remains poorly understood, particularly the roles of the structural restrictions of tubules and collecting ducts, it was suggested that the decrease of FA may result from the necrosis of tubular epithelial cells in medulla, which likely led to water diffusion along the oriented tubular structure impaired [46].

**Figure 3:**
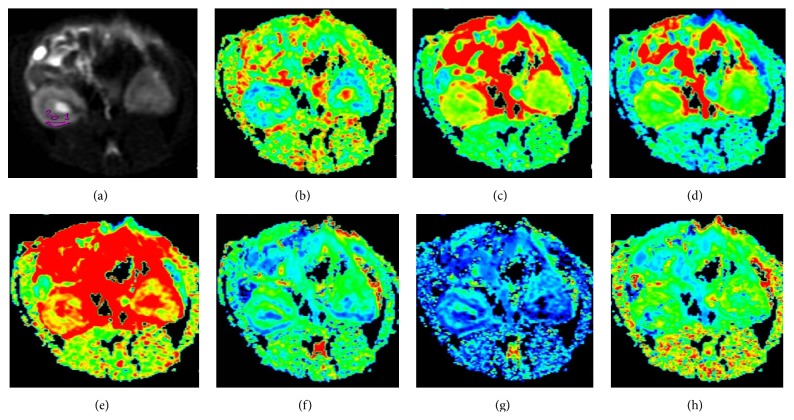
Representative diffusivity and kurtosis maps obtained from diffusional kurtosis imaging for one healthy Sprague Dawley rat. (a) Representative locations of regions of interest (ROIs) for the cortex and medulla at the mid-zone of the right kidney on the *b* = 0 mm^2^/s image. The same ROIs were then copied to maps of all metrics. (b–h) Maps of the fractional anisotropy (FA), mean diffusivity (MD), radial diffusivity (*D*_⊥_), axial diffusivity (*D*_||_), mean kurtosis (MK), radial kurtosis (*K*_⊥_), and axial kurtosis (*K*_||_) are given, respectively.

Due to the presence of structural hindrances in the renal medulla, like membranes or directional structures, the diffusion of water molecules in the kidney is restricted and does not follow a Gaussian distribution. Thus, mathematical models considering the non-Gaussian behavior are needed to more accurately describe the diffusion process. Diffusional kurtosis imaging (DKI), which is a technique based on non-Gaussian water diffusion analysis, has been regarded as an extension of the DTI model that features minor changes in data acquisition and processing [47]. It provides different diffusion parameters, such as the mean kurtosis (MK), radial kurtosis (*K*_⊥_), and axial kurtosis (*K*_||_), which can provide more useful information about the microstructural complexity of tissue. It has been widely applied to brain studies [48, 49]. Pentang et al. [50] and Huang et al. [51] evaluated the feasibility of DKI in normal human kidneys and demonstrated that the kidney is well suited for the application of DKI due to the presence of anisotropy in renal tissue. In our preliminary study (the findings of which have not been published), DKI has also been utilized to study the renal diffusion of healthy Sprague Dawley rat (Figure 3). However, DKI has not been shown to detect changes in non-Gaussian water diffusion in the kidneys due to AKI.

## 5. Conclusion

In summary, functional renal imaging is a growing field of interest with tremendous potential, particularly the BOLD, ASL, and DWI techniques, which assess the oxygenation, perfusion, and diffusion of the kidney. Moreover, because these techniques do not require the administration of exogenous contrast agents, they can also be applied in patients with impaired renal function. Although the lack of standardized sequences, postprocessing software, and models hinders the widespread use of these techniques in clinical settings, numerous published papers have demonstrated the feasibility of the techniques for assessing the renal pathophysiology of AKI triggered by different causes. Further explorations that feature improvements in the hardware and postprocessing software are essential to improve our understanding of the renal pathophysiology and progress of AKI.
